# Tim Winton’s Pneumatic Materialism

**DOI:** 10.1080/1369801X.2020.1715819

**Published:** 2020-02-12

**Authors:** Arthur Rose

**Affiliations:** Department of English, University of Bristol, UK

**Keywords:** Breath, Cloudstreet, Dirt Music, Global South, pneuma, Winton, Tim

## Abstract

The somatic effects of empire can be found in Tim Winton’s “pneumatic materialism”, an aesthetic preoccupation in his novels with moments of anoxia, or the deprivation of oxygen to the brain. This essay will consider how Winton's novel engage with pneumatic materialism in response to questions of uneven development traditionally associated with the Global South, thereby disrupting clear South–North distinctions. By blurring his concerns across the North–South divide, Winton shows a willingness to think of empire as a series of relations that are not bound by national or territorial borders so much as by substances in the air. He does this, I argue, in his use of the breath.

## Introduction

In visual depictions of the Global South, Australia and New Zealand provide a boot-shaped, antipodean exception to the otherwise stable Brandt Line. Yet these countries, too, have their subalterns, disrupting the complacency of a “developed” North all too willing to make uneven development a problem of “over there”. As work by López (2007), Prashad (2012) and Mahler (2015) convincingly demonstrates, the Global South has shifted from its earlier conception as a geospatial term to become a “postglobal discourse”, a “political consciousness” concerned more with the “vulnerable scapegoats” of globalization than with the niceties of geographical boundaries (López 2007, 7; Mahler 2015, 95). The somatic effects of empire manifest even for the supposed beneficiaries in the Global North, demanding new forms of political solidarity. The fiction of Tim Winton has been recognized as dealing imperfectly with the political discontents of the Australian Global South, particularly as they pertain to class, race relations and gender. Indeed, while Winton’s novels are recognized for addressing class questions with nuance and sensitivity, they have been rightly criticized for their marginalization of Indigenous Australians (Rooney 2009) and their problematic depiction of women (Schürholz 2012). In turn, critics have defended the work from these accusations (McGloin 2012; Mathews 2017) on the grounds that characters often seditiously contravene the generic constraints implied by more critical readings and that the occlusions themselves are often marked absences haunting the texts. Neither side of the debate simply focuses on the mimetic effects of formal decisions. Both assume Winton’s formal decisions in creating these effects are directed at quite conventional, liberal causes: the politics of his forms seem simply to index larger conditions.

This essay will disrupt the visual clarity of the Brandt line by considering how Winton’s novels engage with problems “in the air” that refuse to adhere to strict formulas of development and economic security. I argue Winton uses a breath-related formalism – here theorized as a pneumatic materialism – to show how uneven development, traditionally associated with the Global South, might be brought to bear when considering how issues of class, race, environment and mental health affect Western Australia, the region of Winton’s concern. By showing how a conceptual North–South divide functions internally to a nation in the Global North, Winton shows a willingness to think of empire as a series of relations that are not bound by national or territorial borders. Winton’s geographies are more local and more diffuse: aerial, they permeate the bodies of his characters, which are rendered porous, in turn, through this relation with air. Winton enacts this bodily porosity, I argue, in his use of breath.

Winton’s novels betray an aesthetic preoccupation with moments of breathlessness, especially when it leads to a deprivation of oxygen to the brain. While characters have very different encounters with cerebral hypoxia (drowning, long-term lung disease, erotic asphyxiation), each crisis combines a scientific illustration of anoxic effects with a spiritual meditation on the afterlife. Breath provides Winton with the formal means to keep his conflation of hallucination, based on natural science, and revelation, based on divine inspiration, rigorously indeterminate. Winton uses this formal indeterminacy to respond to conditions as various as the occlusion of Indigenous Australians from Australia’s colonial history, the consequences of poaching fish and mining the land for asbestos and bauxite, and the erotic attachment to (auto) asphyxiation.

These anoxic crises do play into a larger, problematic, tendency in postcolonial literature that places “extraordinary bodies of disabled or sick characters” at the center of the narrative (Barker 2016, 100). Drawing on, *inter alia*, Fish from *Cloudstreet*, Clare Barker explains:

As a trope, a narrative device, disability enables postcolonial writers to tell vivid stories about colonialism and its aftermath, stories that resonate outward from a character’s disabled body to address ‘damage,’ inequality, and power and its abuses in the postcolonial world. (2016, 100)

Winton’s anoxic crises do engender damaged bodies, indexing a “postcolonial politics” of air in the wider field of disability studies (Barker 2016, 100). We should be critical of this tendency to instrumentalise disability, as Barker notes. But we can also see how these crises serve to develop a materialist theology that does not reduce Winton’s characters to their disabilities. Moments of pneumatic resurrection draw on a realist context (they are not miraculous) inflected by religious language (the rhetoric often evokes biblical miracle). My challenge in this essay is to connect this materialist theology to Winton’s postcolonial politics, via an aesthetics of breath.

## The aesthetics of breath

Breath, in Winton’s texts, is a theologico-scientific *pneuma*. *Pneuma*, the Greek word for both “breath” and “spirit”, may designate the physical function of respiration or the Christian entity, the Holy Spirit. Between the physical and the spiritual account, however, something “remains” that “exceeds death but that is not yet configured as life,” that which Shelley Rambo has called a “middle space of witness” (2010, 108). Winton uses breathing as a physiological process (respiration) to inaugurate moments of spiritual expression (*pneuma*) to develop, in this “middle space of witness”, what I call “pneumatic materialism”: a spiritual materialism, which is used variously as narrative technique, theme and plot device.

In referring to “pneumatic materialism” I want to extend Lyn McCredden’s reading of Winton’s “poetics of resurrection”, or “a reaching out in literary language for an understanding of the sacred forces of meaning-making hovering within and beyond the human” (2015, 332), to include a more materialist dimension. I agree with McCredden that the “embarrassment” of Winton’s publicly expressed Christian beliefs requires more formal attention (2015, 324), particularly since Winton himself refers to these stylistic elements as attempts “to find a language for human yearning” (quoted in McCredden 2015, 326). Of course, Winton’s work draws on a distinctly religious rhetoric. Like McCredden, I want to consider those moments where the narrative enacts formal features (the vignette) as a consequence of plot events (like drowning). *Pace* McCredden, I will argue that the consequences of Winton’s juxtaposition of the ordinary with the sacred is not a meaning-making extension “beyond the human” (2015, 332). Rather, it configures the sacred as a fragile byproduct of specific materialist functions, namely through visions that do not necessarily equate to a definitive truth claim about the sacred. If Winton is a mind–body dualist, his is an etiolated dualism that relies, principally, on the physiological cause-and-effect of the body, and the “witnessing” reader, for whom the breath-as-process acts as the necessary material for any spiritual enlightenment.

Winton’s formal interest in pneumatic materialism has implications for the ways his novels engage with the “political consciousness” of Australia’s Global South. If McGloin and Mathews are right to read internal discrepancies into the gendered or racial preconceptions, respectively, then a common formal feature (a transversal) across these divergent marked occlusions would be useful for future “intersectional” responses to Winton’s work. My argument is that *pneuma* creates just such conditions, as a common, formal feature of a materialist theology.

In some ways the speed of this theological introduction, however frustrating, allows me to present an interesting aspect of Winton’s limited scientific interest: the materialist interests within his writing seem bent on refuting the post-Cartesian dualism that he himself seems to endorse. But the concern with breath in Winton’s novels is not simply a regression to a simpler pre-scientific respiratory theory. Rather, Winton’s particular form of theological realism begins from the observation of natural phenomena, and precisely the machinic qualities of the breath, from which he extrapolates theological implications.

Pneumatic materialism develops over the course of Winton’s *oeuvre*. It appears in *Cloudstreet* (1998) as a structural device to underpin Winton’s critique of class in his “Great Australian novel”. After 2000, Winton’s pneumatic materialism becomes the means by which he reimagines the lived effects of empire. So, *Dirt Music* (2003) addresses indigenous rights, fishery conservation and asbestos use, while *Breath* (2008) tackles issues like drug addiction and child abuse. In each case, breath is taken as a formalist conceit with historical, political and social consequences. By “formalist conceit” I mean that breath develops a number of poetic features in Winton’s work that distinguishes it from his other themes, characterizations or plots. I do not mean that Winton has an explicit poetics of the breath, in the manner of Paul Celan or Charles Olson.^1^ But, like Celan and Olson, Winton is drawn to the breath as the means by which physiological imperative might be fused to poetic principle. In Winton’s case this is not an explicit theoretical project, as it was in Olson’s *Projective Verse* or Celan’s *The Meridian*. Winton has no correlative intervention on breath as aesthetic principle. And yet, breath, in Winton’s novels, does form the basis for a physiological poesis, and not simply because it is often the means by which theme, character and plot cohere. Breath is a structural element in the novels, a central conceit that drives either the story or the text’s formal innovations. In this sense the narrative is often framed by some breath-related activity, whether the legacy of a cerebral hypoxia (*Cloudstreet*) or the playing of a musical instrument (*Breath*). Even when this framing device is not included, breath features as the impulse behind certain character movements (*Dirt Music*). At the same time, Winton’s signature device, the vignette, might, in its impressionistic brevity, also be understood as breath’s translation into aesthetic form. The vignette is the contracted presentation of a life, impression or scene. It may demand more than a single physiological breath, but it functions as a “breath-unit”, or a discrete conceptual image. Indeed, the vignette requires brevity in order that it might impress upon the reader this unity of expression. Breath figures not simply as poetic function: Winton’s formal concern with breath extends from, and is underpinned by, an observable interest in the effect breath may have on perception, particularly those distortions, permanent or temporary, that accompany cerebral anoxia. Breath also gives rise to important plot turns, in moments of hallucination.

## Distorted perceptions

The interest in distorted perception is observable in *Cloudstreet*, ostensibly the story of a house, called Cloudstreet, and the two working-class families who live in it. The narrative disperses itself across a number of different focal characters, most of whom are members of either the Pickles or Lamb family. The novel charts the period before the families begin to cohabit, then describes, through sporadic vignettes, the vicissitudes of the families’ quasi-separated lives over a twenty-year period. There are a number of characters in and out of whose consciousnesses we drift, as a consequence of Winton’s free indirect discourse; the narration, therefore, coheres around one particular first person narrator, whose occasional foregrounding demonstrates that there is a narrative intent behind the meandering anecdotes of this family saga. This central narrative constitutes itself through the recollections of Fish Lamb, the character whose drowning early in the novel catalyses the Lambs to move to Cloudstreet. Although the Lambs celebrate Fish as returning “back from the dead”, the eldest son, Quick, recognizes that “not all of Fish Lamb had come back” (Winton 1998, 32). In medical terms, Fish has suffered a hypoxic ischemic brain injury with long-term neurological effects, including poor concentration, loss of bowel control, and idiosyncratic language use and development. But Fish is also revealed to be the novel’s narrating consciousness, making the novel an impossible still point in a turning world. If the novel is constituted in a moment of recollection, this is also a moment of crisis: Fish is drowning. The novel, bookended by two descriptions of the moment that Fish goes “to the water” at a family picnic, is an extended flashback of life in Cloudstreet, condensed into the moments before Fish loses consciousness: “Soon you’ll be a man, Fish, though only for a moment, long enough to see, smell, touch, hear, taste the muted glory of wholeness and finish what was begun only a moment ago down there” (420). Barker describes the split as follows:

In medical, realist terms, Fish experiences brain damage that limits his cognitive development, but as a function of the narrative, which allows the surreal and otherworldly to pierce the social realist framework, Fish is granted a kind of split consciousness, whereby the part of him that has not “come back” from near death becomes the novel’s transcendent, philosophical narrative voice. (Barker 2016, 110)

The novel certainly plays with a dualism between a transcendent, philosophical narrative voice and a body that lives with severe learning needs. But it also anticipates a moment of reconciliation between these competing entities: “soon, soon you’ll be yourself, and we’ll be us; you and me. Soon!” (Winton 1998, 420). When this reunion happens, in “the tumble past the dim panic of muscle and nerve into a queer and bursting fullness”, it appears at first that this will be a lasting reunion that reaches into an afterlife (424). However, Winton emphasizes that this is “a hesitation, a pause for a few moments” by iterating the condition “for that long”, “for just that long”, “for those seconds it takes to die, as long as it takes to drink the river, as long as it took to tell you all this” (424). Fish Lamb is not indefinitely reunited with himself. Like Jorge Luis Borges’s Jaromir Hladik in “El milagro secreto”, death defers itself only until after the artwork is complete. After, Fish’s “drinking the river” will reassert itself in real time. What remains, in Shelley Rambo’s terms, is our witnessing this as a material entanglement of breath and spirit.

Rambo meditates on the moment in John 19:30 when Jesus bows his head and, at the moment of his death, “hands over his spirit [*pneuma*]”. Rambo understands this spirit to be neither the Holy Spirit, nor, wholly, the material breath, but something between (“a middle spirit”) implied in the phrase “hands over”. “Handing over” suggests that something remains in death. Death itself is rendered inconclusive, not because of a certain resurrection, but because this middle spirit must be recognized and received in order to complete the act of handing over. So, while the narrative exceeds the terms of Fish Lamb’s death, it does so without fully recognizing the “handing over” of Fish’s spirit to death or resurrection.

The final lines of Fish’s narrative suggest a contrary reading, that the “handing over” has completed itself in a life after: “and then my walls are tipping and I burst into the moon, sun, and stars of who I really am. Being Fish Lamb. Perfectly. Always. Everyplace. Me” (Winton 1998, 424). Does this constitute the life after, the “reaching beyond” of McCredden’s reading? It may well. But I want to test an alternative hypothesis, that these final lines maintain a materialist collision of dualities. In this reading, what he sees (that the walls are tipping, that he bursts into the moon, sun and stars) are simultaneously hallucinations brought on by a lack of oxygen and the self-constituting cosmology of the narrative voice. His perfection, omnipresence and omnitemporality recognize a religious dimension that ultimately comes to rest in the material self-as-object: me. In other words, because it is possible to read Fish’s “time it takes to die” simultaneously as materialist and theological, I suggest this alternative reading, the better to turn the narrative from its allusive metaphysics to the more grounded elements of its physics: the concern it has with water and drowning.

## On water and drowning

Water and drowning are common source metaphors in Winton’s work. There are inverted parallels between the near drowning of the protagonist of *Dirt Music*, Luther Fox, and his memories of his breathless father dying of an asbestos related disease (ARD). The narrator of *Breath*, Bruce Pike, will recollect his own experiences of breath-holding and suffocation after he encounters the body of a boy who has died as a result of erotic autoasphyxiation. Unsurprisingly, associations between water, place and religion in Winton’s work are common in Winton criticism.^2^ Risk, too, has featured prominently in responses to his work. When these accounts merge, drowning is implicated, unsurprisingly, with death, but also with rebirth and resurrection. While death by drowning features as the ultimate consequence of risky behaviour, it also imagines itself with a theological safety net: characters who drown in Winton’s novels are often reborn in an explicitly theological way. As a result, Winton criticism has acknowledged the important thematic role drowning plays in the novels. But this version forgets a critical material feature: the rebirth is never complete; not all of Fish Lamb comes back. Drowning plays a structural role in Winton’s work insofar as it is equally concerned with spiritual allusion and material process.

Drowning, in its medical definition, “is the process of expiring respiratory impairment from submersion/immersion in liquid” (Van Beeck, Branche, and Szpilman 2005, 854). Suominen and Vähätalo gloss this as a “liquid/air interference [that] occurs at the entrance of the airways of the victim, which prevents the victim from breathing air” (2012, 2). Significantly, contemporary medical accounts shift attention away from drowning’s material (liquid) to emphasize its “process” (in this case, the prevention of breathing). Winton describes his own experience of drowning in an interview with Andrew Denton in terms of process:

I was under the boat, trapped. Had fishing line and rope and stuff around my leg and I was kind of drowning. I was sort of in that last moment before, you know … I’m just seeing bubbles, thinking, “Oh, this is beautiful. This is nice”. [At Denton’s prompting, Winton goes on to add] I wasn’t drowning yet. I hadn’t started to inhale any water. I mean, people say that drowning is an easy death. I think that was an old romantic thing that’s … I think it would be horrible. It’d be like suffocating. (Denton and Winton 2004)

By using the term “inhalation,” Winton does indicate a different understanding of drowning to the recognized medical account. For Winton, as for medical researchers, the focus is on process. Where the accounts of drowning differ, then, is in their variable emphasis on experience. The medical account registers the process as mere duration (of immersion, of resuscitation), while Winton imagines comparable experiences: he likens drowning to suffocation. Certainly, feelings of asphyxiation in Winton’s work are often treated in much the same way as liquid immersion.^3^ In *Dirt Music*, written before the interview, and *Breath*, written after, Winton develops metaphoric connections between drowning and suffocation. At the same time, the experience is marked by an aesthetic appreciation of such moments when he sees bubbles and thinks about beauty. Despite an immediate threat of drowning, perhaps even because of it, Winton is thrown into an aesthetic contemplation of the water. Like Fish, in his dual roles as narrator and character, Winton’s moment of crisis is also a moment of pleasant reflection. Hallucination plays an important role in the embodied aesthetic experience of this moment, as Winton indicates earlier in the interview: “You jump in the water and just … It was like a hallucinatory experience, you know? Fish, sharks, dolphins, seals and weird noises, like something out of a Kubrick movie”.^4^ Importantly, this “last moment” of aesthetic or hallucinatory appreciation is the moment before drowning “starts”, which is marked as when one “inhales water” or one begins to suffocate. Winton pairs embodied experience with aesthetic experience, the better to link the two. However, this link anticipates a “fragile future” in which embodied experience will disrupt, even destroy, aesthetic enjoyment.^5^

This unsettling capacity to “disturb and inspire” is very much the matter of the Kubrick film Winton most appreciates: *2001: A Space Odyssey*, where the background to Bowman’s hallucinatory experience at the end of the film, after his systematic murder of the machine HAL 9000, mixes minimalist noises with the sound of breathing. Apart from the hallucinatory elements of *2001*, it is the breath sounds that arrest Winton’s attention:

Kubrick overlays many scenes with the fraught and claustrophobic noise of human respiration, like a mesh of consciousness lacing every apparent abyss, and as a result each mute action is threaded with contingencies so great as to be almost unbearable. (Winton 2016, 25)

The implication is that breath, at least in Winton’s response to Kubrick, threads contingency (“like a mesh of consciousness”) into moments that would otherwise be meaningless (“apparent abyss”). If “Kubrick’s labouring astronauts are most eloquent when they don’t speak … their every breath says all we need to know about their situation”, how then does breath create a fragile mesh of contingency across Winton’s own novels? (Winton 2016, 25–26).

Insofar as the moment of aesthetic contemplation is fragile, it has a necessary relation to risk. Through risk, the moment of aesthetic awareness is both made possible and possibly unmade, a testing which, for Winton, is connected to a feeling of mortality that occurs when testing the limits of the breath:

I was interested in the kind of limits … the limits of things … I think part of that strange male thing in adolescence, to test yourself and to frighten yourself a little bit, was there. We used to dive as deep as we could on one breath. We used to ride waves as big as we could cope. We were always pushing ourselves. And I don’t know what that was about except maybe feeling what it was like to be mortal, you know, feeling what it was like to have things in jeopardy. (Denton and Winton 2004)

If the references to risk and “strange” masculinities resonate with much of the existing criticism, Winton’s interest in the association between breath, “limits” and feelings of mortality, of having things in “jeopardy”, suggests experiences fixed more on breath’s material immediacy than the spirit’s transcendental ambitions “beyond the human”.

When Winton’s account of his near-drowning and his reflections of Kubrick are compared to the formal use of breath in his novels, what emerges is (1) a poetics that draws on hallucination and the vignette to (2) thread together a materialist theology that is (3) fragile and risky. In what follows, I want to recognize the breath-devices that elucidate the formal role played by breath, hallucination and vignette in *Cloudstreet*, *Dirt Music* and *Breath*. Then I want to address how the formally experimental novels incorporate breath as a mediating device. Finally, I conclude by gesturing to the implications this transversal reading of the text might have for a formal intersectional reading of Tim Winton’s political consciousness.

## Breath’s mediations

Cloudstreet, a house on Cloud Street shared by two families, the Pickles and the Lambs, is the object around which the story will turn: the industrious Lambs putting their indolent landlords, the Pickles, to shame with their determination to make the best of the property; the Pickles, unable to sell the house as a stipulation of its inheritance, similarly tied to their situation because of almost unremitting poverty. Critics have noted its parallels to Australian settler origins. The story is told through a series of third person vignettes, though these vignettes do gesture towards free indirect discourse. This gesture becomes somewhat more overt by the time one reaches the end of the novel, when we find out that the novel’s impersonal third person narrator has been Fish Lamb, the unnamed “overripe man’s body” that jumps into the water in the novel’s unmarked preface. Though the reader would be forgiven for not noticing it, the novel that follows is the trace of this unnamed man’s “having known the story for just a moment”. For it works through, in more or less chronological order, how these two families come together for the picnic that brackets the beginning and end of the novel. A catalyst for the Lambs is the death and rebirth of Fish Lamb in an accident with a net while the family is fishing in the local river. As a result of the cerebral anoxia Fish suffers from “drinking in the river”, he sustains permanent brain damage. Yet, as we find out in the novel’s closing, as Fish returns to the river, the narrator has been Fish all along, but only consciously so “for those seconds it takes to die, as long as it takes to drink the river, as long as it took to tell you all this”. A spiritual Fish who reacquaints himself with his brain-damaged animate body as he drowns: “drinking his way into the tumble past the dim panic of muscle and nerve into a queer and bursting fullness”.

In *Cloudstreet* the ambivalence between spiritual and material *pneuma* registers as an opposition of two discourses, exemplified when the Lambs take Fish to a doctor, or “the quack” (Winton 1998, 65). The quack attempts to register the event in terms of causal actions, times taken to recover and the long-term effects. Lester Lamb’s response is to call it a miracle. “Like Lazarus, eh, the quack muttered; Jesus wept” (67). The religious is apparently opposed to the scientific. Although “there is nothing physically wrong with him”, “he’s been alive and he’s been dead … One of those was bound to be a shock” (67). These two sites unite in the water: when he asks Fish where he’d like to go, Fish replies “the water”, signposting his ongoing and urgent desire to finish the process of drowning begun at the beginning of the novel, when he dives into the river, and the beginning of the story, when he drowns for the first time (68).

*Dirt Music*, like *Cloudstreet*, uses anoxia to link narratives together, but rather than a structural link, whereby the form of the novel is, in some ways, explained by the symptoms of hypoxia, it simply explains the rationale of the male protagonist, Luther Fox. If the novel’s milieu is the phenomena of the high-yield fishing industry on Australia’s West Coast, the narrative arc follows Luther Fox as he flees his home, and his lover, Georgie Jutland, who tries to find him. Luther is a poacher who hightails it to the Northern Territories, when it is discovered that he has been poaching and having an affair with the female protagonist, Georgie, who is married to the local fishing hero.

At the midpoint of the novel, Luther arrives in Wittenoom, former home to Australia’s blue asbestos mining industry and the place his father worked. Luther refers to it as “the mine that orphaned him” because his father has died of mesothelioma (Winton 2003, 221). Mesothelioma, an interstitial chest cancer caused by inhaling asbestos fibres, typically affects people twenty to fifty years after exposure. As Luther describes it to Georgie, “He was dyin our whole life. But we didn’t know it” (96). After Wittenoom, Luther will have “no goal, only a vague bearing North” until he fixes on a place that Georgie mentions as a place where she felt she “had always known”, Coronation Gulf (102). The novel’s narrative arcs towards a false climax at Wittenoom – “That’s it? You comes this far and that’s all you wanna see? This is the place that killed his father and five minutes and a dose of hippy piss is all he gives it?” (235) – before continuing North, to the fictional Coronation Gulf, where Luther will attempt to form a relationship with the land that is unmediated by either the recent trauma of losing his family or the more distant trauma of losing his father.

In order to develop this relationship, Winton draws on Luther’s history with music. Once a member of a successful family folk group, Luther has foresworn playing or listening to music. At Coronation Gulf he returns to music, not only to connect to the land but also to sing his history, including his relationship with Georgie. He accompanies himself with a drone, comprising a nylon cord attached to a tree: “He sings until … he wonders whether the tree isn’t bending him now, if he’s the singer or the sung” (Winton 2003, 403). But connecting him across this “resonating multiplication” is the concern with breath. Not simply because the singing-breathing will lead to hallucinations of Georgie “breath[ing] into his mouth” (404), but because his father’s death of mesothelioma connects these moments, the later moments of drowning, and the earlier feelings of drowning through a common symptom: breathlessness.

In the end it was hospital. Lying there like a man being held down in a tub of water. Neck straining at the end of him as though he might get his head out and take a clean breath if only he pushed hard enough. But he was drowning anyway. (Winton 2003, 229)

The symptoms described are linked to the pleural effusions that accompany severe asbestosis and mesothelioma. In the novel, it is the common condition of breathlessness that better explains Luther’s complex response to his own hypoxic situations:

You could stay here, he thinks. On a single breath you could live here on a God-given day like this when plankton spin before your eyes and fish leave their redoubts in phalanxes to swim to you. The thread of heat inside him trickles back to a thudding core. There’s no discomfort now, no impulse to take another breath … He kicks up lazily. From too far and too long down. Poisoned and happy. A distant part of him knows how close he’s come to shallow water blackout, but as he crashes through the glittering surface where his body still does the breathing for him, the rest of him settles for simple ecstasy. He lies half in the world. Tingling. (Winton 2003, 127)

What is this tingling? In Winton’s novels, cerebral hypoxia is linked to hallucinations, euphoria and sexual gratification. This measure between the two worlds, “where his body still does the breathing for him” and where the “plankton spin before your eyes”, and the two bodies, “the distant part of him” who knows about hypoxia and “the rest of him” that “settles for simple ecstasy”, may recall an aforementioned dualism, but it seems to me that both emerge wholly materially in this part of the narrative, an anticipation of not only the moment when Georgie will revive Fox at the end of the novel, but also a transgressive counterpoint to the parallel “drowning” of Fox’s father. Here, the water is *heimat*, or “a site of belonging”, to follow Bill Ashcroft’s reading of the substance (2014, 18). However, it functions more as a conduit for the actual process whereby plot and character are realization, to wit, the breath.

Shifting our focus from the place, water, to the process, breath, is deceptively easy in *Breath*. Like *Cloudstreet*, breath will have a structural dimension, Bruce Pike’s first person narrative being a recollection that is tied to breath activities. At first appearance this activity follows the narrative-driven focus of *Dirt Music*: the parallel anoxias in this novel being erotic asphyxiation, which is variously enjoyed by the suicide victim who opens the novel and the female protagonist, Eva, when she seduces a younger Bruce Pike, and the breath-holding dives of Pike and his friend Loonie, when they practice holding their breaths “so long that our heads were full of stars” (Winton 2008, 15). These thematic parallels are, of course, vital to understanding the novel, since it is through a recognition of the physiological symptoms of erotic asphyxiation that Pike is able to determine other, psychological conditions. There are certainly external parallels with famous cases of erotic auto-asphyxiation related deaths, and the efforts by families to sanitize the scene. Here, I think particularly of the lead singer of INXS, Michael Hutchence, who died under peculiar circumstances in 1997. But rather than explore the more salacious passages of strangulation and suffocation between Pike and Eva, I want to concentrate on the problematic structural use of the didgeridoo, the instrument that, in playing, will frame Bruce Pike’s thoughts. He begins: “I tamp down the beeswax around the pipe and clear my throat. Then I blow until it burns … the wind goes through me in cycles, hot and droning and defiant” (8). That this is a form of recollection emerges ten pages later, when he likens the talking of his childhood to blowing the didgeridoo: “cycling air through and through, doing little more than explaining yourself to your self while you’re still sane enough to do it” (18). And, later, “honking away on my old didj … now the wind comes through me in circles, like a memory, one breath, without pause, hot and long” (43). By the end of the novel we realize that, aside from its unmarked framing sections, the entire novel has been one long breath, tracked by the sound of the didgeridoo: “I blow the didj until it hurts, until my lips are numb, until some old lady across the way gives me the finger” (245).

Pike’s “didj” has been recognized as an appropriation of Indigenous culture. Without apologising for this appropriation, either on the part of Pike or Winton, it is worth placing the use of the didgeridoo in context, since it is the means by which Pike “cycles” through recollections that “if you tried to talk about … you’d be howled down as some kind of nostalgia freak, called a liar before you even got started” (18). Pike begins to play after waking from a dream, where “I’m hanging limp in a faint green light while all the heat ebbs from my chest and the life begins to leach out of me” (7). This drowning moment is interrupted by “someone at the surface, swimming down” (8). The impression that they will “drag me clear, blow air into me hot as blood” is dispelled when he recognizes his own face on the would-be saviour: “my own mouth opens. A chain of shining bubbles leaks forth but I do not understand” (8). The blown air, “hot as blood”, echoes references to the “hot breath” of Luther Fox and Georgie Jutland in their respective efforts to aspirate each other at the end of *Dirt Music*. Pike’s dream repeats the key features of this rescue, as, indeed, it harks back to Winton’s recollections of his own drowning, already mentioned. But if hot breath does function as a necessary restorative, it must also be expelled, in the form of music or narrative, if it is not to stagnate. By this reading, Pike’s appropriation of the didgeridoo simultaneously offers him his only means of self-expression.

It may be that Winton draws on this reference, like the characters Menzies and Axle in *Dirt Music*, and the problematically (un)named “blackfella” of *Cloudstreet* (208), to insist that connections to the land must be routed through Indigenous cultures. Quick’s encounter with the “blackfella” leads him back to the neighbourhood of Cloudstreet, which is just one of the reasons why Peter Mathews identifies the character as “a liminal prophet, corporeal but seeming to possess an occasional otherworldliness, to remind the inhabitants of Cloudstreet about the importance of that place to the two families” (2017, 2). After Luther Fox has visited Wittenoom, it is Menzies and Axle that give him the boat, in order to reach the outer islands of the gulf. It is strangely in these moments, when Winton is appropriating Indigenous cultures and voices, that he arguably makes his most pointed interventions on questions of historical trauma, contemporary mining practices and erotic asphyxiation. As Michael R. Griffiths demonstrates, Fish Lamb allows for an exploration of the ghosts that haunt *Cloudstreet*: “the ghosts that haunt Cloudstreet figure a history of Indigenous prior occupation, as well as colonial dispossession and its form of assimilation” (Griffiths 2014, 79). When Luther attempts to find “somewhere quiet” in *Dirt Music*, Menzies is “doubtful” precisely because, if the territory appears unoccupied, it also has a bauxite deposit, which means “everybody fightin now” (*Dirt Music* 308). The references to Wittenoom have prepared us to imagine that the immediate consequences of these mining claims (“someone gonna kick us off sooner later”) gesture to other, as yet unimagined, harms. In *Breath* the details of Eva’s death (and, indeed, the death of the boy at the beginning of the novel) by autoerotic asphyxiation have been covered over for reasons of shame: “I couldn’t say. I would risk setting off the rolling mass of trouble inside me. I choked it down. At quite some cost” (Winton 2008, 236). If Bruce Pike’s playing allows him “something pointless and beautiful”, it also allows him to air these difficult feelings, to entertain that sense of risk that characterized the social interactions of his younger self, and to reconnect with those places where “I don’t have to be cautious and I’m never ashamed” (247). The use of breath, while problematic, allows for a series of transversal, intersectional responses to problems of history, extraction and shame.

## Conclusion

Anoxic moments in Winton cohere into a novelistic infrastructure. It is an infrastructure that relies on a pneumatic materialism, wherein spirituality is sourced through a materialist understanding of how respiration affects human perception. Identifying pneumatic materialism in Winton’s work has wider implications than as a simple aesthetic response to his form: it develops a network of transversal approaches to Australia’s Global South. When the Black Lives Matter movement took the shocking, final, physiological confession of Eric Garner, “I can’t breathe”, as a rallying cry for Black oppression in the United States, it challenged the so-called Global North to interrogate what constitutes “breathable life”.^6^ This applies as much to Australia, with its entangled histories of mining, oppression and exploitation, as to any other country on the “right side” of the Brandt line. In this light, Winton’s presentation of a breath poetics is not a symptom of his embarrassing religiosity: it is the moral materialism on which he bases his critique of contemporary infringements on “breathable life”.

## Footnotes

1 Celan, who called the moment a poem suspended or shattered its claim to coherence an “atemwende” or “breathturn”, writes in his *Meridian* lecture of the breathturn as the moment when a phrase becomes “a terrifying falling silent, it takes away his – and our – breath and words” (2011, 7). Olson, who sought to link the poetic line more closely with physiological limits imposed by the breath, advocates a “Projective Verse” wherein the rhythm of the poem charts a course from “the HEART, by way of the BREATH, to the LINE” (1966, 19).

2 “For Winton, the rivers and coast of Western Australia around Perth are both evocative of place, and markers of vision and rebirth conveying central tenets of Christian faith” (Murray 2003, 84). Ashcroft takes water to be *Heimat*, a space of belonging, with associations of death, rebirth and redemption (2014, 18).

3 When Quick Lamb is kicked in the chest by a dying kangaroo, he hallucinates that his brother, Fish, is rowing across the wheat fields in a fruit box: “It’s floating up there. I’m under it, thinks Quick, I’m under water, under something. God Almighty, I’m gonna drown” (*Cloudstreet* 200).

4 Winton repeats this sentiment in *The Boy Behind the Curtain* (2016), where experiencing the ocean is again “something out of Kubrick, all hurtling colours and shapes and patterns so intense as to be slightly mind-bending” (125).

5 According to Botha,
fragility exposes a field of anticipation. It addresses itself to an imminent yet indefinite future – a fragile future – which may arrive quite suddenly or be delayed, which may emerge from constructive or destructive processes, which may be reversible or irreversible, and which …  may be characterized by order or disorder. (2014, 3)

6 See Crawley’s discussion of the “otherwise air” of this encounter (2017, 1).
